# Supplementary material for: Inspection of antimicrobial remains in bovine milk in Egypt and Saudi Arabia employing a bacteriological test kit and HPLC-MS/MS with estimation of risk to human health
Source: PLoS One. 2022 Apr 27;17(4):e0267717. doi: 10.1371/journal.pone.0267717 (PMC9045658; doi:10.1371/journal.pone.0267717)
Supplement: Attachment — Submitted filename: PONE-D-22-02795_reviewer.pdf [file pone.0267717.s002.pdf]

PLOS ONE

Inspection of Antimicrobial Remains in Bovine Milk in Egypt and Saudi Arabia Employing a bacteriological test kit and HPLC-MS/MS with Estimation of Risk to Human Health

--Manuscript Draft--

**Manuscript Number:**	PONE-D-22-02795
**Article Type:**	Research Article
**Full Title:**	Inspection of Antimicrobial Remains in Bovine Milk in Egypt and Saudi Arabia Employing a bacteriological test kit and HPLC-MS/MS with Estimation of Risk to Human Health
**Short Title:**	Inspection of Antimicrobial Remains in Bovine Milk
**Corresponding Author:**	Areej M. El-Mahdy, Ph.D.<br>Princess Nourah bint Abdulrahman University<br>Riyadh, SAUDI ARABIA
**Keywords:**	screening; Bovine Milk; Antimicrobial Residues; Oxytetracycline, Bacteriology; HPLC-MS/MS.
**Abstract:**	Veterinary medicine uses antibiotics randomly for treatment and growth promotion. Milk of dairy animals contains substantial quantities of antibiotics that have harmful effects on health. It is therefore necessary to test commercially available milk with immunological, chromatographic, or microbiological methods to avoid antibiotic residues. This study aims to perform a microbiological test, followed by a quantitative confirmation analysis, on raw milk to assess whether antibiotic residues are present. Tests were conducted on 200 milk samples taken from markets and farms in Saudi Arabia and Egypt. The microbial inhibitor test (Delvotest SP-NT) revealed that 40 samples were positive. The positive samples were tested using liquid chromatography-tandem mass spectrometry (LC-MS/MS) as a confirmatory quantitative test for 29 antibiotics that belong to five groups: tetracyclines, sulfonamides, fluoroquinolones, macrolides, and lactamases. Only four samples tested positive for oxytetracycline residues above the maximum residue limit. Based on these results, researchers suggest a monitoring system that takes into account both microbial and HPLC-MS/MS methods when detecting antibiotic residues in Bovine milk. The analysis of risk to human health revealed that antibiotic remains in found concentrations do not pose any health risks to consumers.
**Order of Authors:**	Nora H. Al-Shaalan
	Jenny Jeehan Nasr Nasr
	Shereen Shalan Shalan
	Areej M. El-Mahdy, Ph.D.
**Additional Information:**	
**Question**	**Response**
**Financial Disclosure**<br><br>Enter a financial disclosure statement that describes the sources of funding for the work included in this submission. Review the submission guidelines for detailed requirements. View published research articles from *PLOS ONE* for specific examples.<br><br>This statement is required for submission	This research was funded by The Deanship of Scientific Research at Princess Nourah bint Abdulrahman University, through the Research Funding Program (Grant No# FRP-1440-26)

Powered by Editorial Manager® and ProduXion Manager® from Aries Systems Corporation

and **will appear in the published article** if the submission is accepted. Please make sure it is accurate.<br><br>**Unfunded studies**<br>Enter: *The author(s) received no specific funding for this work.*<br><br>**Funded studies**<br>Enter a statement with the following details:<br>• Initials of the authors who received each award<br>• Grant numbers awarded to each author<br>• The full name of each funder<br>• URL of each funder website<br>• Did the sponsors or funders play any role in the study design, data collection and analysis, decision to publish, or preparation of the manuscript?<br>• **NO** - Include this sentence at the end of your statement: *The funders had no role in study design, data collection and analysis, decision to publish, or preparation of the manuscript.*<br>• **YES** - Specify the role(s) played.<br><br>* typeset	
**Competing Interests**<br><br>Use the instructions below to enter a competing interest statement for this submission. On behalf of all authors, disclose any competing interests that could be perceived to bias this work—acknowledging all financial support and any other relevant financial or non-financial competing interests.<br><br>This statement is required for submission and **will appear in the published article** if the submission is accepted. Please make sure it is accurate and that any funding sources listed in your Funding Information later in the submission form are also declared in your Financial Disclosure statement.<br><br>View published research articles from *PLOS ONE* for specific examples.	The authors have declared that no competing interests exist

Powered by Editorial Manager® and ProduXion Manager® from Aries Systems Corporation

**NO authors have competing interests**<br><br>Enter: *The authors have declared that no competing interests exist.*<br><br>**Authors with competing interests**<br><br>Enter competing interest details beginning with this statement:<br><br>*I have read the journal's policy and the authors of this manuscript have the following competing interests: [insert competing interests here]*<br><br>* typeset	
**Ethics Statement**<br><br>Enter an ethics statement for this submission. This statement is required if the study involved:<br><br>• Human participants<br>• Human specimens or tissue<br>• Vertebrate animals or cephalopods<br>• Vertebrate embryos or tissues<br>• Field research<br><br>Write "N/A" if the submission does not require an ethics statement.<br><br>General guidance is provided below. Consult the submission guidelines for detailed instructions. **Make sure that all information entered here is included in the Methods section of the manuscript.**	N/A

**Format for specific study types**<br><br>**Human Subject Research (involving human participants and/or tissue)**<br>• Give the name of the institutional review board or ethics committee that approved the study<br>• Include the approval number and/or a statement indicating approval of this research<br>• Indicate the form of consent obtained (written/oral) or the reason that consent was not obtained (e.g. the data were analyzed anonymously)<br><br>**Animal Research (involving vertebrate animals, embryos or tissues)**<br>• Provide the name of the Institutional Animal Care and Use Committee (IACUC) or other relevant ethics board that reviewed the study protocol, and indicate whether they approved this research or granted a formal waiver of ethical approval<br>• Include an approval number if one was obtained<br>• If the study involved *non-human primates*, add *additional details* about animal welfare and steps taken to ameliorate suffering<br>• If anesthesia, euthanasia, or any kind of animal sacrifice is part of the study, include briefly which substances and/or methods were applied<br><br>**Field Research**<br><br>Include the following details if this study involves the collection of plant, animal, or other materials from a natural setting:<br>• Field permit number<br>• Name of the institution or relevant body that granted permission	
**Data Availability**<br><br>Authors are required to make all data underlying the findings described fully available, without restriction, and from the time of publication. PLOS allows rare exceptions to address legal and ethical concerns. See the PLOS Data Policy and FAQ for detailed information.	Yes - all data are fully available without restriction

A Data Availability Statement describing where the data can be found is required at submission. Your answers to this question constitute the Data Availability Statement and **will be published in the article**, if accepted.<br><br>**Important:** Stating 'data available on request from the author' is not sufficient. If your data are only available upon request, select 'No' for the first question and explain your exceptional situation in the text box.<br><br>Do the authors confirm that all data underlying the findings described in their manuscript are fully available without restriction?	
**Describe where the data may be found in full sentences. If you are copying our sample text, replace any instances of XXX with the appropriate details.**<br><br>• If the data are **held or will be held in a public repository**, include URLs, accession numbers or DOIs. If this information will only be available after acceptance, indicate this by ticking the box below. For example: *All XXX files are available from the XXX database (accession number(s) XXX, XXX.).*<br>• If the data are all contained **within the manuscript and/or Supporting Information files**, enter the following: *All relevant data are within the manuscript and its Supporting Information files.*<br>• If neither of these applies but you are able to provide **details of access elsewhere**, with or without limitations, please do so. For example:<br><br>*Data cannot be shared publicly because of [XXX]. Data are available from the XXX Institutional Data Access / Ethics Committee (contact via XXX) for researchers who meet the criteria for access to confidential data.*<br><br>*The data underlying the results presented in the study are available from (include the name of the third party*	If the data are all contained within the manuscript and/or Supporting Information files, enter the following: All relevant data are within the manuscript and its Supporting Information files.

and contact information or URL).<br>• This text is appropriate if the data are owned by a third party and authors do not have permission to share the data.<br><br>* typeset	
Additional data availability information:	Tick here if your circumstances are not covered by the questions above and you need the journal’s help to make your data available.

Powered by Editorial Manager® and ProduXion Manager® from Aries Systems Corporation

Manuscript
Click here to access/download;Manuscript;Revised Manuscript.docx

Research Paper

Inspection of Antimicrobial Remains in Bovine Milk in Egypt and Saudi Arabia Employing a bacteriological test kit and HPLC-MS/MS with Estimation of Risk to Human Health

Nora H. Al-Shaalan[1], Jenny Jeehan Nasr[2], Shereen Shalan[2], Areej M. El-Mahdy[3,4,*]

[1] Chemistry Department, College of Science, Princess Nourah bint Abdulrahman University, Riyadh 11671, Saudi Arabia.

[2]Department of Pharmaceutical Analytical Chemistry, Faculty of Pharmacy, Mansoura University, Mansoura 35516, Egypt.

[3]Department of Pharmaceutical Sciences, College of Pharmacy, Princess Nourah bint Abdulrahman University, Riyadh 11671, Saudi Arabia.

[4]Department of Microbiology and Immunology, Faculty of Pharmacy, Mansoura University, Mansoura 35516, Egypt

____

*Author for correspondence. Areej M. El-Mahdy, Tel (00966565657400), E-mail: amalkasaby@pnu.edu.sa

Abstract

Veterinary medicine uses antibiotics randomly for treatment and growth promotion. Milk of dairy animals contains substantial quantities of antibiotics that have harmful effects on health. It is therefore necessary to test commercially available milk with immunological, chromatographic, or microbiological methods to avoid antibiotic residues. This study aims to perform a microbiological test, followed by a quantitative confirmation analysis, on raw milk to assess whether antibiotic residues are present. Tests were conducted on 200 milk samples taken from markets and farms in Saudi Arabia and Egypt. The microbial inhibitor test (Delvotest SP-NT) revealed that 40 samples were positive. The positive samples were tested using liquid chromatography-tandem mass spectrometry (LC-MS/MS) as a confirmatory quantitative test for 29 antibiotics that belong to five groups: tetracyclines, sulfonamides, fluoroquinolones, macrolides, and lactamases. Only four samples tested positive for oxytetracycline residues above the maximum residue limit. Based on these results, researchers suggest a monitoring system that takes into account both microbial and HPLC-MS/MS methods when detecting antibiotic residues in Bovine milk. The analysis of risk to human health revealed that antibiotic remains in found concentrations do not pose any health risks to consumers.

Keywords: Screening; Bovine Milk; Antimicrobial Residues; Oxytetracycline, Bacteriology; HPLC-MS/MS.

1. Introduction

Antibiotics are a broad group of medicines that are utilized to destroy or prevent bacterial microorganisms from developing. In the field of human medicine, they are used to treat a variety of microbial infections. In the field of animal husbandry, they are widely used for infection treatment, but they are also illegally used for prophylaxis and growth promotion, which can boost a farm's financial return [1,2]. Utilizing these products may deliver residues into milk, triggering unfavorable allergic reactions in humans [3]. Moreover, antimicrobials may cause antibiotic-resistant bacteria to flourish, which can cause serious medical conditions [4]. Various governments have established monitoring projects to establish antibiotic concentrations in food and a maximum residue limit (MRL) for them [5,6]. Consequently, the European Union (EU) has prohibited the illegal growth promotion usage of antibiotics. Global organizations and State governing agencies like the World Health Organization (WHO) and the United Nations Food and Agriculture Organization (FAO) (collectively formed the Codex Alimentarius), and the United States Department of Agriculture (USDA) have established MRLs for medicines intended in veterinary usage and their presence is allowed in nutrients of animal source [7]. In developing countries, however, veterinary drug abuse is detected at shocking rates due to inadequate supervision and limited analytical controls [8].

Checking nutrients of animal sources for the existence of antibiotic residues is generally executed by screening procedures, which comprise microbiological tests, and confirmatory quantitative procedures including liquid chromatography coupled to mass spectrometry [9]. Microbiological tests contain two major groups: tube and multi-plate tests. The multi-plate test consists of dishes holding agar medium with diverse indicator bacteria. Specimens are placed on the top of the agar surface, then, after incubation, the growth of bacteria will change the opacity of

the agar. If antibiotic residues are contained in the specimen, inhibition of the growth of bacteria will occur, producing a clear zone nearby the specimen. A disadvantage of this test is that it is time-consuming due to the continuous need for fresh agar and fresh bacterial cultures; hence, they are inappropriate for macroscale [10].

Tube tests, which are commercially accessible, employed for onsite screening of antibiotic residues. They consist of ready-to-use tubes comprising microorganisms on focus together with the nutrients, indicator, and an agar medium [11]. The tube methods are used efficiently for the examination of residual antibiotic drugs in the livestock food than the multi-plate as it is neither time-consuming nor exhausting [12]. The most common indicator bacterium used in these inhibition tests is *Geobacillus stearothermophilus*, because of several reasons such as low contamination level, tolerance to high temperature (55°C), and shorter incubation time (less than 4 h) compared to other bacteria. Furthermore, it is more susceptible to antibiotics, especially, β-lactams [13].

For example, the Delvotest® SP NT (produced by DSM Food Specialties Ltd., The Netherlands), which is a standard diffusion test for the screening of antibacterial substances in dairy milk through inhibiting the growth of *Bacillus stearothermophilus* strain, that is susceptible to several antibiotics and sulfa drugs. During the growth of bacteria, they generate acid, thus, altering the agar pH. The existence of antibiotic traces detected merely by comparing colors. This kit requires only 100 µL of the milk sample and only 3 hours incubation. Thus, Delvotest® SP NT presented as an appropriate, speedy, simple to use, and low-cost alternative test for the detection of numerous antibiotics in milk products. It is also suitable for the simultaneous analysis of a large number of specimens applying a short and simple process. The previous literature reported several analytical procedures describing screening of antibiotic remains in milk [14,15]. These practices

utilized microbial assays and instrumental analysis. Even though analytical methods as HPLC induce precise data of the concentration of residual antibiotics, it needs costly apparatus and skilled investigators. Because of the speed and easiness of microbiological assays, they are applicable for the prescreening of probable antibiotics. Nevertheless, objectionable false negative or false-positive findings might arise if using microbiological testing.

The probable unfavorable impacts on human health produced by antimicrobial residues determined by computing the risk estimation. Normally, estimation of chemical risk involves four distinct steps; hazard recognition, hazard description/dose-response estimation, exposure evaluation, and risk depiction. Estimation of chemical risk has two extensively utilized concepts, which are; Hazard quotient (HQ) and risk quotient (RQ). The hazard quotient is utilized for the estimation of health risk whilst the risk quotient is employed in the assessment of environmental risk. This methodology is greatly favored for maintaining food safety to guarantee public health [15].

The current study aimed to perform prescreening of raw milk specimens to detect antibiotic residues by microbiological inhibitor test kits followed by confirmatory quantitative analysis by an HPLC/MS-MS technique. Furthermore, antibiotic residues in milk were correlated with risk estimation for human health. In this study, the novelty is the application of a microbiological method followed by confirmation chromatography for screening antibiotic residues in milk samples in the Arab region, including Egypt and Saudi Arabia for the first time, and the assessment of health risk associated with positive results.

2. Materials and methods

2.1. Milk samples

Two hundred bovine milk samples were gathered from different locations from Saudi Arabia and Egypt after approval from Institutional Review Board, Princess Nourah bint Abdulrahman University (IRB Log Number: 21-0296) as shown in (Table 1). Samples collected from Saudi Arabia were 100 samples from various markets in Riyadh. One hundred milk samples from Egypt were collected from five different Governorates (Cairo, Giza, Dakahlia, Gharbia, Sharkia, Kafr El Sheikh, and Qalyoubia). All milk samples from Saudi Arabia were pasteurized milk of different brands, while Egyptian milk samples were both raw and pasteurized milk of different brands. All samples were kept at 4 °C and analyzed by microbiological test.

Table 1. Collection sampling plan for local raw and pasteurized milk products

Sample type	Country	Sampling region	No. of samples
**Local raw milk**	Egypt	Cairo Governorate	10
		Giza Governorate	10
		Dakahlia Governorate	10
		Gharbia Governorate	10
		Sharkia Governorate	10
		Kafr El-Sheikh Governorate	10
		Qalyoubia Governorate	10
**Local pasteurized milk**	Egypt	Juhayna products	5
		Lamar Egypt products	5
		Dina Farms products	5
		Almarai products	5
		Beyti products	5
		Lactel products	5
**Local pasteurized milk**	Saudi Arabia	**Almarai products**	
		Full cream milk	5
		Low-fat milk	5
		Full cream laban	5
		Low-fat laban	5

Ayran laban	5
**Alsafi products**	
Full cream milk	5
Low-fat milk	5
Skimmed milk	5
Full cream laban	5
Low-fat laban	5
**Nadec products**	
Full cream milk	5
Low-fat milk	5
Full cream laban	5
Low-fat laban	5
**Saudia products**	
Full cream milk	5
Low-fat milk	5
**Activia products**	
Full cream milk	5
Low-fat milk	5
Full cream laban	5
Low-fat laban	5
**Total Samples**	**200**

2.2. Delvotest SP-NT (Microbial inhibitor test)

Delvotest SP-NT is a non-specific microbiological test, performed to identify the existence of antibiotic residues in dairy milk. The principle of the test is agar diffusion, in which the agar test tubes contain a fixed standard count of *Bacillus stearothermophilus* spores, nutrient agar, and bromocresol purple as a pH indicator. Delvotest SP NT purchased from DSM Food Specialties located in Spain [16]. The sample was added directly to the agar surface (ampoules), then incubated at 64 °C for 3 h. After incubation, color is changed from purple to yellow due to a change in pH resulting from microbial metabolism. In the case of fermented milk, heating first for 10 min at 80 °C to remove natural inhibitors lysozyme and lactoferrin [17]. Test and data interpretation

carried out based on the manufacturer's instructions. Visual interpretation of the test results evaluated as 'negative' (yellow) and 'positive' (blue or purple).

2.3. Chemicals

The antibiotic analytical standards; tetracycline HCl, oxytetracycline HCl, chlortetracycline HCl, doxycycline hyclate, nalidixic acid, norfloxacin, ofloxacin, enrofloxacin, ciprofloxacin, lomefloxacin HCl, erythromycin A, oleandomycin triacetate, Tylosin tartrate, tilmicosin, Josamycin, spiramycin, roxithromycin, lincomycin HCl, clindamycin, trimethoprim, sulfadiazine, sulfadoxine, sulfamethoxazole, sulfathiazole, sulfachlorpyridazine, sulfanilamide, and sulfamethoxazole, were obtained from Sigma–Aldrich (Seelze, Germany). The Arab Company for Gelatin and Pharmaceutical Products (Alexandria, Egypt) provided ampicillin and amoxicillin raw materials. Analytical grade Formic acid, disodium hydrogen phosphate dihydrate, citric acid monohydrate, and trichloroacetic acid were of analytical grade and were purchased from Merck (Darmstadt, Germany). Phosphoric acid was from Riedel-deHaën (Seelze, Germany). Preparation of the McIlvaine buffer was by adding 0.1 M citric acid hydrate to 0.2 M disodium hydrogen phosphate (60:40, v/v). The washing solution was prepared by blending water and methanol (95:5, v/v). Twenty percent trichloroacetic acid solution was made to precipitate protein. Regenerated cellulose membrane filters and syringe filters (Minisart RC25) with pore size 0.45 µm were from Sartorius-Stedim (Goettingen, Germany). The solid-phase extraction columns Chromabond ABC18 (C18) sorbent were purchased from Macherey-Nagel, Düren, Germany.

2.4. Equipment

Confirmatory chromatographic analysis was executed on an Agilent Technologies HPLC system 1260 (Agilent Technologies, USA). Detection using mass spectrometry was undertaken employing a triple quadrupole API 4500 (ABSciex, Canada), that operated in the positive electrospray ionization under selected reaction monitoring mode. The mass spectrometer settings used were as follows: dwell-time = 20 ms; resolution Q1 and Q3 = unit; nebulizer gas = 12 psi; curtain gas = 12 psi; collision gas = 8 psi; ion spray voltage = 5500 V; temperature = 400 ◦C. Controlling the hardware and the data procurement and treatment were accomplished utilizing Analyst 1.6.3 Software (ABSciex, Canada). A vortex shaker from Heidolph (Schwabach, Germany), a TDL-60B Centrifuge (Anke, Taiwan), and BHA-180 T Sonicator (Abbotta Corporation, USA) were employed for the sample preparation and the extraction procedure.

2.5. Chromatographic Conditions

All conditions used were according to the reference method. LC analyses were executed using a Nucleodur MN-C18 column (150 mm × 4.6 mm i.d., 5 µm particle size), Macherey-Nagel, Düren, Germany. The mobile phase used was a gradient of parts A (water containing 0.2% formic acid) and B (acetonitrile containing 0.2% formic acid) at an oven temperature of 30°C with a flow rate of 0.3 mL/min. The gradient started with 90% of eluent A for 1 min, then reduced to 40% through 11 min. This composition was kept steady for 3 min, then raised to 90% of eluent A within 1 min.

2.6. Stock Standard Solutions

Preparation of stock standard solutions of all analytical standards was performed by accurately weighing the materials that were dissolved in methanol. But, for solubilization of quinolones, it is necessary to add 2% of a 2 M ammonium hydroxide solution to methanol solution. All stock solutions of concentration 1 mg/mL were stored in the refrigerator. Working standard mixed solutions of each group of antibiotics were prepared in a concentration of 20-fold MRL for tetracyclines, macrolides, lincosamides, and quinolones and 10-fold MRL for sulfonamides through dilution with the mobile phase (water and acetonitrile 90:10, v/v, with 0.2% formic acid). If no MRL existed, the concentration of the analyte in the mixture was 0.2 μg/mL.

2.7. Preparation of Samples

Preparation of milk samples was performed adopting a reference method [18], 5 mL of each of suspected milk samples were weighed in a centrifuge tube. The samples were mixed with 100 μl of trichloroacetic acid solution 20% (w/v) and the mixtures were vortexed. 10 mL of McIlvaine buffer of pH 4.0 were added and the mixtures were vortexed for 1 min and then, subjected to centrifugation at 4000 rpm for 15 min. The supernatant was removed and filtered. The filtrate was then exposed to a solid-phase extraction procedure. First, conditioning of the SPE cartridges was made using 6 mL of methanol and 6 mL of water. The filtrate was then moved to the cartridge. The washing step was done with 6 mL of 5% methanol in water (v/v), the cartridges were then dried for 10 min. Then, elution of the analytes was done using 6 mL methanol, followed by vaporization to dryness in nitrogen. The residue was redissolved in 1 mL of the mobile phase (water and acetonitrile 90:10, v/v, with 0.2% formic acid) and analyzed.

2.8. Calculation of Hazard Quotient and Risk Estimation

The model of Hazard Quotient was utilized to estimate the risk of ingesting residues with milk. Hazard quotient is defined as the ratio of the prospective exposure to a material and the concentration where no harmful impacts are anticipated.

$$Hazard\ Quotient = \frac{Estimated\ Daily\ Intake\ (EDI)}{Acceptable\ Daily\ Intake\ (ADI)}$$

The estimated daily intake (EDI) was computed using the subsequent equation presented by Juan et al. [19].

$$EDI = \frac{Concentration\ of\ Residue\ in \frac{\mu g}{\text{[illegible]}} \times Daily\ Intake\ of\ milk\ in\ Kg/person}{Adult\ Body\ Weight\ (Kg)}$$

The mean level of residual antibiotics in raw milk was calculated. Then, the mean concentration and normal daily milk consumption based on 60 kg body weight were utilized for calculations. According to the data supplied by the Food and Agriculture Organization of the United Nations, The per capita availability of milk in Egypt was 96.98 ml/day [20].

Acceptable Daily Intake (ADI) is an approximated quantity of residue permitted to be consumed daily throughout a life expectancy with no noticeable health risk stated based on body weight. ADI of oxytetracycline is 0.03 mg/kg bw/day [21].

If the hazard quotient is lower than or equal to one, this implies insignificant hazard whilst if the value is greater than one, it indicated the possibility of harm [22].

3. Results

3.1. Delvotest SP-NT (Microbial inhibitor test)

In this study, the detection of antibiotic residues in milk samples was performed using a microbial inhibitor test and liquid chromatography-mass spectrometry. Results of Delvotest SP-NT revealed that 40 out of 200 tested samples showed no color change or partial color change, suggesting a positive result constituting 20 % of the total samples as shown in (Fig 1). After that, all of the 40 positive samples were tested using HPLC-MS/MS method as a confirmatory test.

Fig.1. Results of Microbial inhibitor test (Delvotest SP-NT)

3.2. HPLC-MS/MS

Results of HPLC-MS/MS revealed that only 4 out of 40 suspected samples analyzed were found truly positive (10 %) containing oxytetracycline residues. We could not find any of the other tested antibiotics from the rest of the suspected positive samples via HPLC-MS/MS analysis which were consequently, considered false positive. These positive results were detected in milk samples obtained from the farms in Egypt. The four positive samples contained 120, 132, 141, and 150 µg/Kg, thus, exceeding the MRL which is 100 µg/Kg [20]. It could be concluded that milk obtained from treated cows contained a residual antibiotic. The Chromatogram obtained because of the analysis is presented in (Fig 2).

Fig.2. Typical chromatogram of milk sample positive for oxytetracycline.

3.3. Hazard Quotient and Risk Estimation

The estimation of health risk for confirmed positive milk samples from dairy farms was conducted to verify the threats to consumers resulting from the intake of milk containing antibiotic residues as they were exceeding the MRL. The mean oxytetracycline concentrations in the tested milk samples were 120, 132, 141, and 150 μg/kg. HQ for detected oxytetracycline remains in milk samples from dairy farms was computed to estimate any health threats to consumers (Table 2). As the HQ for the detected residues of oxytetracycline in milk samples from farms was less than one, so it may be presumed that there were insignificant adverse impacts on the consumer health accompanying the intake of the investigated samples.

Table 2. Estimation of Human Health Risk based on hazard quotient for oxytetracycline residues through milk consumption in adults and children from dairy farms

Mean Concentration (μg/Kg)	Age Group	Body Weight[a] (Kg)	EDI	ADI (*3*)	Hazard Quotient
120	Adults	60	0.194	30	0.0065
120	Children	10	1.164	30	0.0388
132	Adults	60	0.213	30	0.0071
132	Children	10	1.280	30	0.0427
141	Adults	60	0.228	30	0.0076
141	Children	10	1.368	30	0.0456
150	Adults	60	0.242	30	0.0081
150	Children	10	1.455	30	0.0485

Abbreviations: ADI, acceptable daily intake; EDI, estimated daily intake.

[a]Body weights for different age groups were taken from FAO/WHO guidelines.

4. Discussion

Examining milk samples for detection of antibiotic residues in levels more than was set by community legislation needs low-cost testing methods. Due to their effectiveness, microbial inhibition methods were largely used instead of physical-chemical methods. Those methods have many advantages as they could be easily operated with no need for special training, simple equipment, and could detect a broad variety of antibiotic traces within a single test [23]. The most commonly used tests are microbiological tests using *Bacillus stearothermophilus* spores, Delvotest SP, Copan Test, Charm Farm-960 Test, and others [16]. In the current study, the test that is used for the detection of the antibiotic in the examined milk samples collected from both KSA and Egypt is Delvotest SP-NT. Non-specificity is the main limitation of these microbial assays [17]. Results of Delvotest SP-NT were assessed visually as purple and yellow colors, which are easy to visualize. However, the problem was for reading the samples containing intermediate concentrations of antibiotics that render the visual reading of the reaction more difficult [24,25]. In those samples, the agar medium appeared as a mix of purple in a yellow background indicating a doubtful positive result. Moreover, different milk types and different antibiotic mechanisms of action make it more challenging to assess due to their different colors [25]. Therefore, microbiological testing is less appropriate for conclusive analyses leading to debatable results (false positives). Another illustration of false-positive Delvotest results may occur due to incomplete milking and natural inhibitors in samples from freshly cows [26]. These results should therefore be confirmed by more specific and sensitive techniques, such as HPLC-MS/MS, which was used in this study to confirm the Delvotest results. In this study, the results of Delvotest SP-NT revealed that the positive samples were those collected from treated farm cows. HPLC-MS/MS

also confirmed that samples obtained from farms were positive for antibiotic residues. This may be due to collection of milk just after antibiotic administration, drug misuse, or bad hygiene [27-30]. HPLC-MS/MS revealed only four positive samples containing oxytetracycline residues as was reported in previous articles [31]. The low number of milk samples containing antibiotic residues may be due to the use of growth promoters by farmers rather than the use of antibiotics. The results of this study coincide with studies in Algeria that were done to detect antibiotics in milk. Hakem et al [32], detected no antibiotic residues in milk samples obtained from two Dairies Mitidja's Farms using Delvotest SP NT. In another study, about 10 % of bulk tank milk samples and 20 % of untreated bovine milk were reported positive [33]. These results were higher than those observed in Algiers (9. 87 %) provided by Ben-Mahdi & Ouslimani [34]. On the other hand, other studies ran against ours including, Zinedine et al.[27] in Morocco, Tarzaali et al. [35] in Mitidja, Aggad et al. [36] in the west of Algeria and Titouche et al. [37] in Tizi-Ouzu. Those detected a higher percentage of antibiotic residues in milk (57, 89, 29, and 46 %, respectively). The HPLC-MS results we obtained were close to those reported by Martins et al. [38] in Brazil, who found 1.76 % of antibiotic residues in milk samples. Other studies produced a positive ratio of more than 15 % as reported by Li et al. [39] in China and García et al. [40] in Spain reporting 28 %.

To determine the threat posed to consumers by milk containing antibiotic residues at levels exceeding the MRL, health risk estimations were conducted for confirmed positive milk samples collected from dairy farms. The mean oxytetracycline concentrations in the tested milk samples were 120, 132, 141, and 150 µg/kg. HQ for detected oxytetracycline remains in milk samples from dairy farms was computed to estimate any health threats to consumers (Table 2). HQ is defined as the ratio of the possible exposure to a material and the concentration where no harmful impacts

are noted. If HQ is less than or equal to one, the risk to human health will be insignificant, while if HQ is exceeding one, the consumer is subjected to the risk. As the HQ for the detected residues of oxytetracycline in milk samples from farms was less than one, so it may be presumed that there were insignificant adverse impacts on the consumer health accompanying the intake of the investigated samples. Comparable results were stated by Moudgil et al. [41], who assessed the dietary exposure to residual antibiotics detected in raw and commercial milk samples in Punjab, India. The study stated no toxicological threat to consumers accompanying the intake of the examined milk samples concerning the antibiotics under study. Similar conclusions were also described by Rahman et al. [22], where the estimated dietary exposure to residual antibiotics through milk in Bangladesh was lower than the toxicological standard value.

5. Conclusion

One of the most significant concerns affecting public health is antibiotic residues found in milk. According to this study, Egyptian and Saudi Arabian cow's milk contained low levels of antibiotics. Among 40 suspicious samples examined, only four (10%) tested positive for oxytetracycline residues. From the remaining suspected positive samples, none of the other examined antibiotics were detected by HPLC-MS/MS, and so this was deemed a false positive. These positive results were detected in samples obtained from the farms in Egypt. The four positive samples contained 120, 132, 141, and 150 µg/Kg, thus, exceeding the MRL which is 100 µg/Kg. Hereby, the milk collected from treated cows contained antibiotic residues. The occurrence of antibiotic residues in milk indicates the importance of further control of milk, which is tested using microbiological Delvotest SP-NT and confirmed with HPLC-MS/MS. We could

conclude that HPLC-MS/MS could be considered a reliable analytical method for determining whether milk contains multiple antibiotics.

Funding

This research was funded by The Deanship of Scientific Research at Princess Nourah bint Abdulrahman University, through the Research Funding Program (Grant No# FRP-1440-26)

Conflict of interest

The authors did not declare any conflicts of interest.

References

1. Yamaguchi T, Okihashi M, Harada K, Konishi Y, Uchida K, Do MHN, et al. Antibiotic residue monitoring results for pork, chicken, and beef samples in Vietnam in 2012-2013, J. Agric. Food Chem. 2015; 63: 5141–5145. doi:10.1021/jf505254y.
2. Peris-Vicente J, García-Ferrer D, Mishra P, Albiol-Chiva J, Durgbanshi A, Carda-Broch S, et al. Procedure for the Screening of Eggs and Egg Products to Detect Oxolonic Acid, Ciprofloxacin, Enrofloxacin, and Sarafloxacin Using Micellar Liquid Chromatography, Antibiotics. 2019; 8: 226.
3. Chen J, Ying GG, Deng WJ. Antibiotic Residues in Food: Extraction, Analysis, and Human Health Concerns, J. Agric. Food Chem. 2019; 67: 7569–7586. doi:10.1021/acs.jafc.9b01334.
4. Ben Y, Fu C, Hu M, Liu L, Wong MH, Zheng C. Human health risk assessment of antibiotic resistance associated with antibiotic residues in the environment: A review, Environ. Res. 2019; 169: 483–493.

5. United States Department of Agriculture. Maximum Residue Limits (MRL) Database | USDA Foreign Agricultural Service, (n.d.). https://www.fas.usda.gov/maximum-residue-limits-mrl-database (accessed April 15, 2021).
6. Tadesse T. Public Health Impacts of Antibiotic Residues in Foods of Animal Origin: A Review. www.iiste.org (accessed April 15, 2021), 2017.
7. Food and Agriculture Organization of the United Nations. CODEXALIMENTARIUS FAO-WHO, (n.d.). http://www.fao.org/fao-who-codexalimentarius/en/ (accessed May 12, 2021).
8. Angulo FJ, Collignon P, Wegener HC, Braam P, Butler CD. The Routine Use of Antibiotics to Promote Animal Growth Does Little to Benefit Protein Undernutrition in the Developing World, Clin. Infect. Dis. 2005; 41: 1007–1013.
9. Stead S, Stark J. Bioanalytical Screening Methods, in: Chem. Anal. Antibiot. 2011; Residues Food, John Wiley and Sons, 153–186.
10. Ngoc Do MH, Yamaguchi T, Okihashi M, Harada K, Konishi Y, Uchida K, et al. Screening of antibiotic residues in pork meat in Ho Chi Minh City, Vietnam, Using a microbiological test kit and liquid chromatography/tandem mass spectrometry, Food Control. 2016; 69: 262–266. doi:10.1016/j.foodcont.2016.05.004.
11. Pikkemaat MG. Microbial screening methods for detection of antibiotic residues in slaughter animals, Anal. Bioanal. Chem. 2009; 395: 893–905.
12. Chung HH, Bin Lee J, Chung YH, Lee KG. Analysis of sulfonamide and quinolone antibiotic residues in Korean milk using microbial assays and high performance liquid chromatography, Food Chem. 2009; 113: 297–301.
13. de Zayas-Blanco F, García-Falcón MS, Simal-Gándara J. Determination of sulfamethazine in milk by solid phase extraction and liquid chromatographic separation with ultraviolet

detection, Food Control. 2004; 15: 375–378.

14. Ramírez A, Gutiérrez R, Díaz G, González C, Pérez N, Vega S. et al. High-performance thin-layer chromatography-bioautography for multiple antibiotic residues in cow's milk, J. Chromatogr. B Anal. Technol. Biomed. Life Sci. 2003; 784: 315–322.
15. Joint FAO/WHO Expert Committee on Food Additives. 2016. Module II: Scientific guidelines for the preparation of veterinary drug residue monographs, working papers and related summary documents for Joint FAO/WHO Expert Committee on Food Additives (JECFA) drafting experts and reviewers assigned by FAO. http://www.fao.org/3/b1003e/b1003e.pdf (accessed July 17, 2021).
16. Žvirdauskienė R, Šalomskienė J. An evaluation of different microbial and rapid tests for determining inhibitors in milk. Food Control. 2007; 18: 541–7.
17. Bilandzic N, Kolanovic BS, Varenina I, Scortichini G, Annunziata L, Brstilo M, et al. Veterinary drug residues determination in raw milk in Croatia. Food Control. 2011; 22: 1941–8.
18. Bohm DA, Stachel CS, Gowik P. Multi-method for the determination of antibiotics of different substance groups in milk and validation in accordance with Commission Decision 2002/657/EC, J. Chromatogr. A. 2009; 1216: 8217–8223.
19. Juan C, Moltó JC, Mañes J, Font G. Determination of macrolide and lincosamide antibiotics by pressurised liquid extraction and liquid chromatography-tandem mass spectrometry in meat and milk, Food Control. 2010; 21: 1703–1709. doi:10.1016/J.FOODCONT.2010.05.004.
20. Food and Agriculture Organization of the United Nations. Maximum Residue Limits (MRLs) and Risk Management Recommendations (RMRs) For Residues Of Veterinary Drugs in Foods CX/MRL 2-2018, n.d.

21. Australian Pesticides and Veterinary Medicines Authority. Acceptable daily intakes for agricultural and veterinary chemicals | Australian Pesticides and Veterinary Medicines Authority, (n.d.). https://apvma.gov.au/node/26596#O (accessed July 17, 2021).
22. Rahman M, Hassan M, Chowdhury S. Determination of Antibiotic Residue in Milk and Assessment of Human Health Risk in Bangladesh, Preprints. 2020. doi:10.20944/PREPRINTS202006.0221.V1.
23. Pikkemaata MG, Rapallinib MLBA, Oostra-van Dijka S, Elferinka JWA. Comparison of three microbial screening methods for antibiotics using routine monitoring samples. Anal Chim Acta. 2009; 637: 1–2
24. Suhren G, Luitz M. Evaluation of microbial inhibitor tests with indicator in micro-titer plates by photometric measurements. Milchwissenschaft. 1995; 50: 467–70.
25. Stead SL, Ashwin H, Richmond SF, Sharman M, Langeveld PC, Barendse JP, et al. Evaluation and validation according to international standards of the Delvotest® SP-NT screening assay for antimicrobial drugs in milk. Int Dairy J. 2008; 18: 3–11.
26. Hillerton JE, Halley BI, Neaves P, Ros MD. Detection of antimicrobial substances in individual cow and quarter milk samples using delvotest microbial inhibitor tests. J Dairy Sci. 1999; 82(4): 704–11.
27. Zinedine A, Faid M, Benlemlih M. Détection des résidus d’antibiotiques dans le lait et les produits laitiers par méthode microbilogique. REMISE. 2007; 1: 1–9.
28. Petrović JM, Katić VR, Bugarski DD. Comparative examination of the analysis of β-lactam antibiotic residues in milk by enzyme, receptor–enzyme, and inhibition procedures. Food Anal Methods. 2008; 1: 119–25.
29. Mensah SEP, Aboh AB, Salifou S, Mensah GA, Sanders P, Abiola FA, et al. Risques dus aux

résidus d'antibiotiques détectés dans le lait de vache produit dans le Centre Bénin J Appl Biosci. 2014b; 80(1).

30. Boultif L. Détection et quantification des résidus de terramycine et de pénicilline dans le lait de vache par chromatographie liquide haute performance (HPLC). Thèse de Doctorat: Université Mentouri Constantine, Algérie. 2015.
31. Furusawa N. Rapid liquid chromatographic determination of oxytetracycline in milk. J. Chromatogr. A. 1999; 839: 247–251.
32. Hakem A, Yabrir B, Khelef D, Laoun A, Mouffok F, El-gallas N, et al. Evaluation of microbial quality of raw milk into two dairies Mitidja's Farms (Algeria). Bulletin USAMV Vet. Med. 2012; 69: 1–2.
33. Layada S, Benouareth D, Coucke W, Andjelkovic M. Assessment of antibiotic residues in commercial and farm milk collected in the region of Guelma (Algeria). Int. J. Food Contam. 2016; 3: 19
34. Ben-Mahdi M, Ouslimani S. Mise en évidence des résidus d'antibiotiques dans le lait de vache produit dans l'Algérois. Eur J Sci Res. 2009; 36(3): 357–62.
35. Tarzaali D, Dechicha A, Gharbi S, Bouaissa MK, Yamnaine N, Guetarni D. Recherche des résidus des tétracyclines et des bêta-lactamines dans le lait cru par le MRL Test (ROSA TEST) à Blida, Algérie. Papier présenté dans : 6èmes Journées Scientifiques Vétérinaires sur le médicament vétérinaire : nouvelles approches thérapeutiques et impact sur la santé publique. (Search for tetracycline and beta-lactam residues in raw milk by the MRL Test (ROSA TEST) in Blida, Algeria. Paper presented in: 6th Veterinary Scientific Days on veterinary medicinal products: new therapeutic approaches and impact on public health) E.N.V, Algérie. 2008; 23–24. French

36. Aggad H, Mahouz F, Ammar YA, Kihal M. Evaluation de la qualité hygiénique du lait dans l'ouest algérien. (Evaluation of the hygienic quality of milk in western Algeria.). Revue de Méd Vét. 2009; 160(12): 590–5. French

37. Titouche Y, Hakem A, Houali K, Yabrir B, Malki O, Chergui A, et al. Detection of antibiotics residues in Raw Milk produced in freha area (Tizi-Ouzou), Algeria. Bulletin UASVM, Vet. Med. 2013; 70: 1843–5378.

38. Martins MT, Melo J, Barreto F, Hoff RB, Jank L, Bittencourt MS, et al. A simple, fast and cheap non-SPE screening method for antibacterial residue analysis in milk and liver using liquid chromatography– tandem mass spectrometry. Talanta. 2014; 129: 374–83.

39. Li H, Xia X, Xue Y, Tang S, Xiao X, Li J, et al. Simultaneous determination of amoxicillin and prednisolone in bovine milk using ultra-high performance liquid chromatography tandem mass spectrometry. J Chromatogr B. 2012; 900: 59–63

40. García ND, Junza A, Zafra-Gomez A, Barron D, Navalon A. Simultaneous determination of quinolone and β-lactam residues in raw cow milk samples using ultrasound-assisted extraction and dispersive-SPE prior to UHPLC-MS/MS analysis. Food Control. 2016; 60: 382–93.

41. Moudgil P, Bedi JS, Aulakh RS, Gill JPS. Analysis of antibiotic residues in raw and commercial milk in Punjab, India vis-à-vis human health risk assessment, J. Food Saf. 2019; 39:g e12643.

Supporting information

S1 Fig. Results of Microbial inhibitor test (Delvotest SP-NT)

S2 Fig. Typical chromatogram of milk sample positive for oxytetracycline.

S1 Table. Collection sampling plan for local raw and pasteurized milk products

S2 Table. Estimation of Human Health Risk based on hazard quotient for oxytetracycline residues through milk consumption in adults and children from dairy farms

 Click here to access/download;Figure;Fig 1. tiff.tif

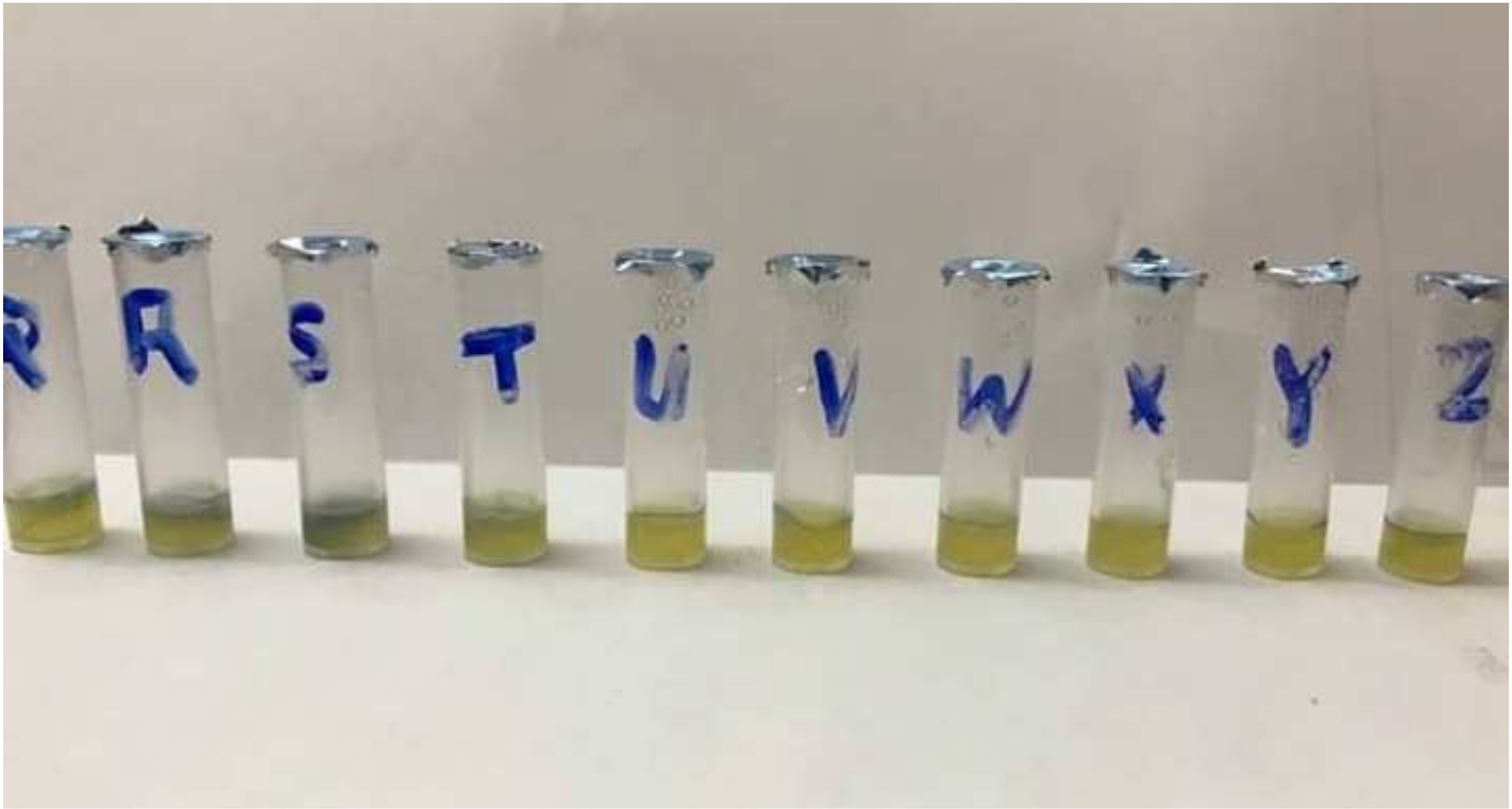

Figure

Click here to access/download;Figure;Fig 2. tiff.tif

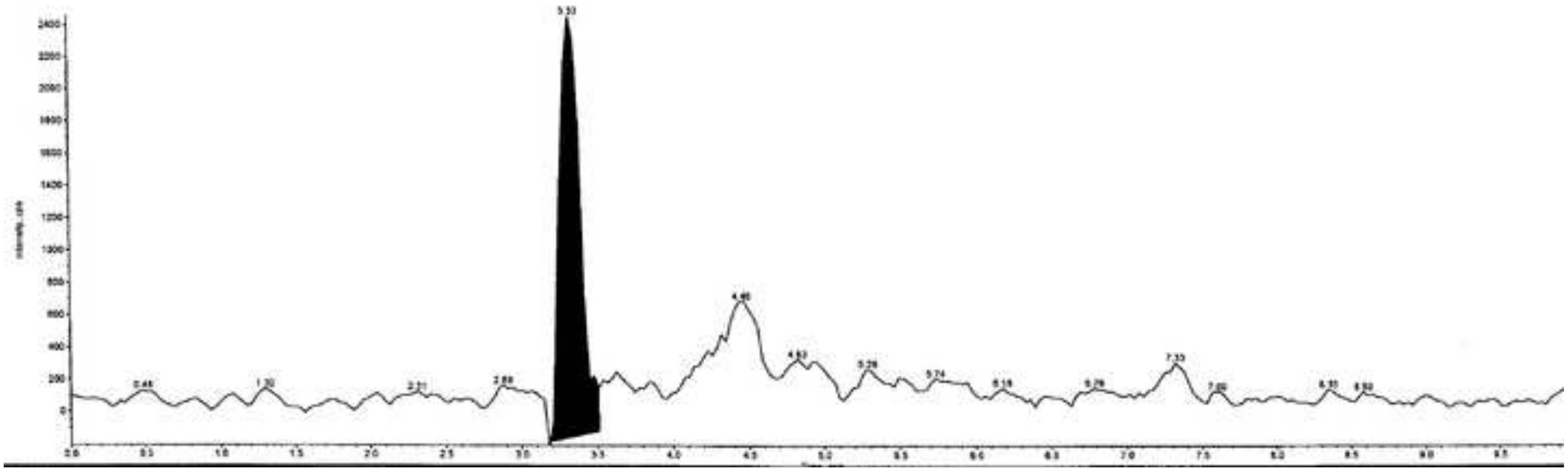

Click here to access/download
Supporting Information
Supporting information.docx

Dear editor:

I have incorporated all of your suggestions into my revision. They were very helpful. Thank you.

- Proofreading and copy-editing was done.
- The novelty of this work was added.
- Information about LC-MS/MS setting was also added
