# Supplementary material for: Inspection of antimicrobial remains in bovine milk in Egypt and Saudi Arabia employing a bacteriological test kit and HPLC-MS/MS with estimation of risk to human health
Source: PLoS One. 2022 Apr 27;17(4):e0267717. doi: 10.1371/journal.pone.0267717 (PMC9045658; doi:10.1371/journal.pone.0267717)
Supplement: Attachment — Submitted filename: Response to reviewers.docx [file pone.0267717.s001.docx]

**Dear editor:**

I have incorporated all of your suggestions into my revision. They were very helpful. Thank you.

- Proofreading and copy-editing was done.
- The novelty of this work was added.
- Information about LC-MS/MS setting was also added
