# Supplementary material for: Inspection of antimicrobial remains in bovine milk in Egypt and Saudi Arabia employing a bacteriological test kit and HPLC-MS/MS with estimation of risk to human health
Source: PLoS One. 2022 Apr 27;17(4):e0267717. doi: 10.1371/journal.pone.0267717 (PMC9045658; doi:10.1371/journal.pone.0267717)
Supplement: Attachment — Submitted filename: Response to reviewers.docx [file pone.0267717.s003.docx]

**Dear editor:**

Thanks for your suggestions. They were very helpful. They were all incorporated in my revision.

- Figure files were added to the Preflight Analysis and Conversion Engine (PACE) digital diagnostic tool.
- For the minimal data set underlying the results, all relevant data are provided in the manuscript.

**Dear Reviewer 1:**

I have incorporated all of your suggestions into my revision. They were very helpful. Thank you.

1- The manuscript was re-edited by Dr. Hanan Shehata, Postdoctoral fellow, University of Guelph.
2- All your valuable comments were done and it will be in the revised manuscript with track changes.

**Dear Reviewer 2:**

- Thank you for your valuable comments.
- All of your comments were done and it will be in the revised manuscript with track changes.
